# Construction of Supramolecular Nanostructures from V-Shaped Amphiphilic Rod-Coil Molecules Incorporating Phenazine Units

**DOI:** 10.3390/polym9120685

**Published:** 2017-12-07

**Authors:** Junying Xu, Shengsheng Yu, Keli Zhong, Long Yi Jin

**Affiliations:** 1 Key Laboratory for Organism Resources of the Changbai Mountain and Functional Molecules, Department of Chemistry, College of Science, Yanbian University, Yanji133002, China; 2105010389@ybu.edu.cn (J.X.); 2016001019@ybu.edu.cn (S.Y.); 2 College of Chemistry, Chemical Engineering and Food Safety, Bohai University, Jinzhou 121013, China

**Keywords:** bent-shaped, aggregation behavior, nanostructures, self-assembly, molecular interaction

## Abstract

A series of bent-shaped molecules, consisting of dibenzo[*a,c*]phenazine and phenyl groups connected together as a rod segment, and poly(ethylene oxide) (PEO) with a degree of polymerization (DP) of 6 as the coil segment, were synthesized. The self-assembling behavior of these molecules by differential scanning calorimetry (DSC), thermal optical polarized microscopy (POM), small-angle X-ray scattering spectroscopy (SAXS), atomic force microscopy (AFM), and transmission electron microscopy (TEM), revealed that carboxyl or butoxy carbonyl groups at the 11 position of dibenzo[*a,c*]phenazine noticeably influence self-organization of molecules into supramolecular aggregates in bulk and aqueous solutions. Molecules **1** and **2** with chiral or non-chiral PEO coil chains and the carboxyl group at the rod segments self-organize into a hexagonal perforated lamellar structure and a hexagonal columnar structure in the solid state. In aqueous solution, molecules **1** and **2** self-assemble into diverse lengths of nanofibers, whereas molecules **3** and **4** with butoxy carbonyl groups exhibit a self-organizing capacity to form diverse sizes of spherical aggregates.

## 1. Introduction

Elaborate design of molecules and the creation of supramolecular nanostructures with well-defined shapes and sizes offer new strategies for the development of nanoclusters and molecular electronics, biomimetic chemistry, and material science [1,2,3].Among the architecture of molecular self-assembly, π-conjugated amphiphilic molecular assemblies have attracted a great deal of attention due to their notable potential as advanced highly-functionalized materials in the area of nanoscience [4,5,6,7,8]. In this context, amphiphilic rod-coil molecular systems have been used for the construction of supramolecular assemblies, and have been widely applied in biomimetic chemistry and drug-delivery systems [9,10,11]. The formation of precise nanostructures from rod-coil molecules requires the design of suitable associative forces, such as π–π stacking, hydrogen bonding, electrostatic interactions, hydrophobic and hydrophilic interactions, and so forth [12,13,14]. As a typical instance of self-assembled materials, by modifying the molecular structures of the aromatic core and the flexible coil chains, the rod-coil molecules are able to form a variety of ordered supramolecular nanostructures, such as spherical, columnar, lamellar, vesicular and fibrous structures, in bulk state and solution [15,16,17]. For example, Lee et al. and other research groups have reported the self-assembling behavior of variously shaped rod-coil molecules, including dumbbell-shaped, T-shaped, bent-shaped, Y-shaped, propeller-shaped, and cyclic rod-coil molecules [18,19,20,21]. They constructed one-dimensional (1-D) lamellar, two-dimensional (2-D) columnar, three-dimensional (3-D) hexagonal and tetragonal structures of supramolecular assemblies in crystalline or liquid-crystal states and created helical fibers, toroids, helical tubules, 2-D flat sheets, open porous sheets, and temperature-sensitive tubules with an expansion-contraction motion in selective solution states [22,23]. 

**Scheme 1:**
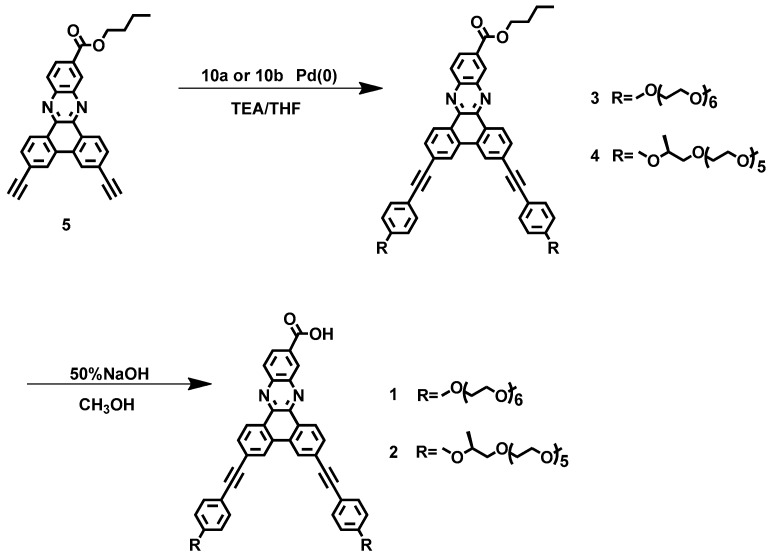
Molecularstructures of **1**–**4**.

Over the past decade, many studies have been performed on the self-organization of bent-shaped block copolymers [24,25,26,27]. For example, Tschierske and co-workers reported the synthesis and phase behavior of some bent-core molecules formed by a bent aromatic core, oligo(siloxane) units, alkyl segments and constructed undulated smectic and oblique columnar nano-structures in the liquid crystalline phase [28,29,30,31,32,33]. Recently, we have also reported the synthesis and self-assembling behavior of T-shaped and bent-shaped amphiphilic molecules, incorporating a dibenzo[*a*,*c*]phenazine unit at the rod segments [34,35,36]. The molecules containing methyl or alkyl groups at the surface of the coil and rod segments, or in the center of the rod building block, spontaneously organized into oblique columnar, lamellar structures and 3-D body-centered tetragonal structures, respectively, through changes in the modified rod-coil molecular structures. In aqueous solution, the bent-shaped molecule self-assembled into micelle, cylindrical or helical nanostructures, by controlling the self-assembling driving force of the molecules. These results implied that the shape of the rod building block, and the lateral groups in the center of the rod segments or at the surface of the rod and coil domains, dramatically affects the arrangement of the rod-coil molecules in the bulk state and in aqueous solution [37]. This is realized by tuning the intermolecular interaction of the bent-shaped molecules to form various supramolecular nanoaggregations. However, as far as we know, there is little literature on the self-assembly of bent-shaped amphiphilic rod-coil molecules incorporating the dibenzo[*a*,*c*]phenazine unit. Therefore, in this study, we designed and synthesized a series of amphiphilic bent-shaped molecules, **1**–**4**, consisting of dibenzo[*a*,*c*]phenazine and phenyl groups connected together as a rod segment and poly(ethylene oxide) (PEO) with a degree of polymerization (DP) of 6 as the coil segment (Scheme 1). These molecules contain carboxyl or butoxy carbonyl groups at the 11 position of dibenzo[*a*,*c*]phenazine and incorporate lateral methyl group at the surface of the rod and coil segments. The molecular aggregation behavior of **1**–**4** was studied by using DSC, POM, DLS, SAXS, TEM and AFM.

## 2. Materials and Methods

### 2.1. Materials

9,10-phenanthrenequinone(Alfa Aesar, Shanghai, China), pyridine(Sinopharm Chemical Reagent Co., Ltd., Shanghai, China), 4-iodophenol(Alfa Aesar, Shanghai, China), potassium carbonate(Sinopharm Chemical Reagent Co., Ltd., Shanghai, China), glacialaceticacid(Alfa Aesar, Shanghai, China), butanol, liquid bromine (Alfa Aesar, Shanghai, China), nitrobenzol (Alfa Aesar, Shanghai, China), benzoyl peroxide (BPO) (Alfa Aesar, Shanghai, China), 3,4-diaminobenzoic acid methyl ester (Alfa Aesar, Shanghai, China), ethylene glycol (Aladdin, Shanghai, China), triethylamine (TEA) (Aladdin, Shanghai, China), trimethylsilylacetylene (TMSA) (Alfa Aesar, Shanghai, China), tetrakis(triphenylphosphine) palladium(0) (Alfa Aesar, Shanghai, China), copper iodide (CuI) (Alfa Aesar, Shanghai, China), iodophenol (Alfa Aesar, Shanghai, China), toluene-*p*-sulfonyl chloride (TsCl) (Alfa Aesar, Shanghai, China) and other reagents were used as received. Tetrahydrofuran (THF) (Sinopharm Chemical Reagent Co., Ltd., Shanghai, China) was treated with sodium metal to remove trace water using benzophenone (Alfa Aesar, Shanghai, China) as chromogenic reagent. 

### 2.2. Techniques

The synthesized molecules were purified by flash column chromatography using silica gel as stationary phase (200–300 mesh). ^1^H NMR spectra were measured on a Bruker AM-300 spectrometer (Bruker, Karlsruhe, Germany) in CDCl_3_ (Beijing Chongxi High-Tech Incubator Co., Ltd., Beijing, China) using TMS (Alfa Aesar, Shanghai, China) as internal reference. MALDI-TOF mass spectra were obtained via a Perseptive Biosystems Voyager-DESTR (matrix: 2-cyano-3-(4-hydroxyphenyl) acrylic acid (CHCA)) (Acros, Geel, Belgium). DSC was performed on a Perkin-Elmer Pyris (Perkin Elmer, Waltham, MA, USA). The heating and cooling rates were controlled to 10 °C·min^−1^ under N_2_ atmosphere. SAXS spectra (Beijing Accelerator Laboratory, Beijing, China) were obtained from the 1W2A X-ray beam line at Beijing Accelerator Laboratory, using synchrotron radiation in transmission mode at various temperatures [38]. POM textures of samples were provided by the Carl-Zeiss polarized optical microscope (Carl Zeiss, Jena, Germany), equipped with a Linkam hot-stage. The UV-VIS absorption and the FL emission spectra were recorded on Shimadzu UV-1650PC spectrometer (Shimadzu, Tokyo, Japan) and a Hitachi F-4500 fluorescence spectrometer (Shimadzu, Tokyo, Japan), respectively. TEM observations were carried out by using JEM-2100F (JEOL, Tokyo, Japan) at 80 KV. Atomic force microscope (AFM) measurements were performed using Agilent 5500 SPM (Agilent Technologies Inc., Santa Clara, CA, USA). DLS measurements were performed using ZetasizerNano-ZS (Malvern, Malvern, UK).

### 2.3. Synthesis of Molecules ***3*** and ***4***

Molecules **3** and **4** were synthesized using the same procedure. A representative example is described for molecule **3**. Butyl 3,6-diethynyldibenzo[*a,c*]phenazine-11-carboxylate (0.19 g, 0.45 mmol) (see Supplementary Materials, Scheme S4) and 19-(4-iodophenoxy)-2,5,8,11,14,17-hexaoxanonadecane (0.49 g, 0.99 mmol) (see Supplementary Materials, Scheme S1) were dissolved in the mixed solution of dry TEA (30 mL) and THF (20 mL). Then, tetrakis(triphenylphosphine) palladium(0) (225 mg, 1.20 mmol) and CuI (682 mg, 0.59 mmol) were added under dark conditions. The mixture was heated to reflux under nitrogen for 48 h. The solvent was removed in a rotary evaporator, and the resulting mixture was poured into water and extracted with dichloromethane, and dried over anhydrous magnesium sulfate. The crude product was purified by column chromatography using ethyl acetate/methanol (50:1 and then 10:1 *v*/*v*) as eluent to yield luminous yellow liquid (0.13 g, 23.8%). ^1^H NMR (300 MHz, CDCl_3_, δ, ppm): 9.16 (d, *J* = 3.0 Hz, 2H), 8.94 (d, *J* = 3.0 Hz, 1H), 8.37 (d, *J* = 6.0 Hz, 2H), 8.34 (d, *J* = 3.0 Hz, 1H), 8.34 (d, *J* = 3 Hz, 1H), 7.76 (d, *J* = 3 Hz, 2H), 7.58 (d, *J* = 6.0 Hz, 4H), 6.96 (d, *J* = 6.0 Hz, 4H), 4.47–4.43 (m, 2H), 4.23 (s, 4H), 3.92 (s, 4H), 3.86–3.57 (m, 40H), 3.39 (s, 6H), 1.86 (t, *J* = 6.0 Hz, 2H), 1.57 (d, *J* = 6.0 Hz, 2H), 1.06 (t, *J* = 6.0 Hz, 3H). MALDI-TOF-MS: *m/z* [M]^+^ 1168.

Molecule **4**: luminous yellow liquid (0.096 g, 19.3%). ^1^H NMR (300 MHz, CDCl_3_, δ, ppm): 9.16 (d, *J* = 3.0 Hz, 2H), 8.94 (d, *J* = 3.0 Hz, 1H), 8.54 (d, *J* = 6.0 Hz, 2H), 8.35 (d, *J* = 3.0 Hz, 1H), 8.22 (d, *J* = 3.0 Hz, 1H), 7.76 (d, *J* = 6.0 Hz, 2H), 7.58 (d, *J* = 6.0 Hz, 4H), 6.96 (d, *J* = 6.0 Hz, 4H), 4.47–4.43 (m, 2H), 4.20 (s, 2H), 3.92 (s, 4H), 3.76–3.57 (m, 40H), 3.39 (s, 6H), 2.06–1.86 (m, 2H), 1.60–1.55 (m, 8H), 1.08 (t, *J* = 6.0 Hz, 3H). MALDI-TOF-MS: *m/z* [M]^+^ 1196.

### 2.4. Synthesis of Molecules ***1*** and ***2***

Molecule **3** (0.05 g, 0.04 mmol) was dissolved in the mixed solution of methanol (40 mL) and 50% NaOH (0.07 mL) aqueous solution, and the mixture was heated to reflux under nitrogen for 24 h. Then, HCl was added dropwise to adjust the PH to about 6, and stirring was continued for 0.5 h. The solvent was removed using rotary evaporators, and water was added into the resulting mixture; then, the organic compounds were extracted with dichloromethane, and dried using anhydrous magnesium sulfate. The obtained yellow solid of crude compound was purified by column chromatography to produce 40.0 mg ( 83.3%) bright yellow viscous solid (stationary phase: aluminum oxide; eluent: mixed solvent of petroleum ethyl acetate and methanol, 30:1 and then 10:1). ^1^H NMR (300 MHz, CDCl_3_, δ, ppm): 9.35 (d, *J* = 3.0 Hz, 2H), 9.02 (s, 1H), 8.69 (s, 1H), 8.42 (d, *J* = 3.0 Hz, 1H), 8.33 (d, *J* = 3.0 Hz, 1H), 7.86 (d, *J* = 6.0 Hz, 2H), 7.66–7.57 (m, 4H), 7.46 (s, 1H), 6.96 (d, *J* = 3.0 Hz, 4H), 4.19 (t, *J* = 3.0 Hz, 4H), 3.92 (t, *J* = 3.0 Hz, 4H), 3.88–3.57 (m, 40H), 3.38 (s, 6H). MALDI-TOF-MS: *m/z* [M + Na]^+^ 1135.

Molecules **2**: a bright yellow viscous solid (0.04 g, 87.7%). ^1^H NMR (300 MHz, CDCl_3_, δ, ppm): 9.28 (d, *J* = 9.0 Hz, 1H), 9.09 (d, *J* = 9.0 Hz, 1H), 8.87 (s, 1H), 8.30 (s, 1H), 8.19 (s, 1H), 8.07 (s, 1H), 7.79 (d, *J* = 3.0 Hz, 1H), 7.76 (d, *J* = 3.0 Hz, 1H), 7.70 (d, *J* = 3.0 Hz, 4H), 7.39 (d, *J* = 3.0 Hz, 1H), 6.95 (d, *J* = 3.0 Hz, 4H), 5.30 (s, 2H), 4.69 (s, 2H), 3.69–3.55 (m, 40H), 3.37 (s, 6H), 1.37 (s, 6H). MALDI-TOF-MS: *m/z* [M + Na]^+^ 1163.

## 3. Results and Discussion

The synthetic route of amphiphilic bent-shaped molecules **1**–**4**, consisting of dibenzo[*a*,*c*]phenazine and phenyl groups connected together as a rod segment and poly(ethylene oxide) (PEO) with a degree of polymerization (DP) of 6 as the coil segment, is outlined in Scheme 1. Rod-coil molecules **1**–**4** were successfully produced via substitution reaction, Sonogashira coupling reaction and hydrolysis reaction, using 9,10-phenanthrenequinone, 3,4-diaminobenzoic acid methyl ester, ethylene glycol, trimethylsilylacetylene and iodophenol as starting materials. The molecular structures were characterized by^1^H NMR and MALDI-TOF mass spectroscopy, and the results were in accordance with the structures shown in Scheme 1 (see Supplementary Materials, Figures S1–S9).

**Figure 1:**
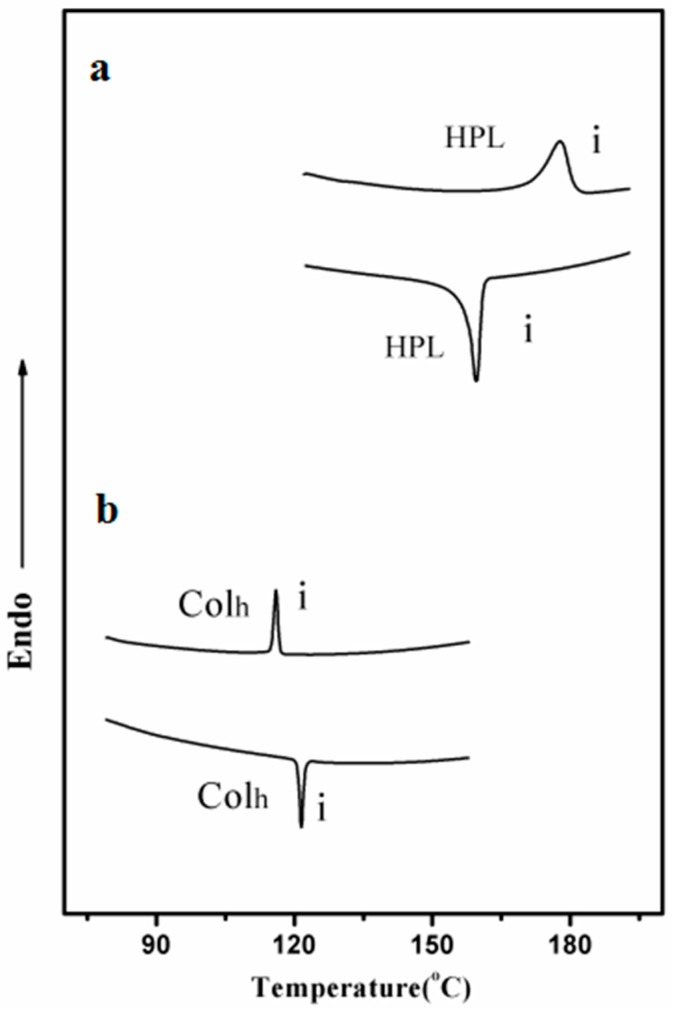
DSC traces (10 °C·min^−1^) recorded during the heating and cooling scans of (**a**) **1** and (**b**) **2** (HPL: hexagonal perforated lamella; i: isotropic phase; Col_h_: hexagonal columnar).

The ordered nanostructures of molecules **1** and **2** were investigated by DSC, POM and SAXS in bulk state. To determine the phase transition temperatures of these molecules, we performed a DSC experiment (see Figure 1), and summarized the resulting data in Table 1.

**Figure 2:**
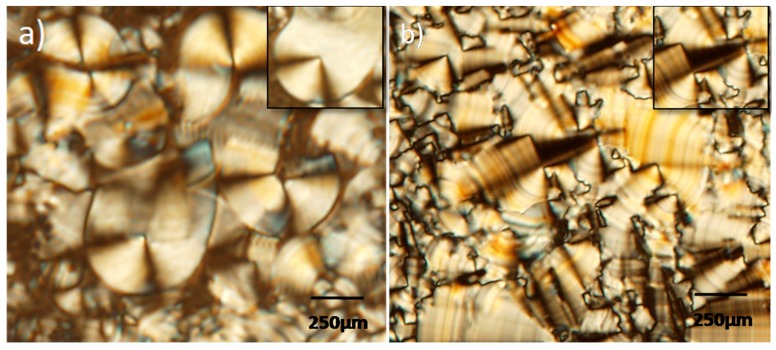
Representative optical polarized micrographs (40×) of the texture exhibited by (**a**) **1** at 158 °C and (**b**) **2** at 106 °C during the cooling.

As is clearly shown in the Table 1, the melting transition temperature of molecule **1** is higher than that of molecule **2**. This is due to the presence of methyl groups between the rod and coil segments, leading to the loose stacking of molecule **2**. Interestingly, in comparsion with molecules **1** and **2**, molecules **3** and **4**, with a butoxycarbonyl group in the 11 position of the dibenzo[*a*,*c*]phenazine, show liquid phase at room temperature. This result further implies that lateral alkoxy groups at the dibenzo[*a*,*c*]phenazine rod building block significantly affect the phase transition temperatures of molecules. On slow cooling of **1** and **2**, from the isotropic liquid to crystalline mesophase, a focal conical fan texture with arced striations and a spherulitic fan texture are observed by POM experiment, respectively, which could preliminarily suggest the presence of hexagonal symmetry (Figure 2) [40,41,42,43].

**Figure 3:**
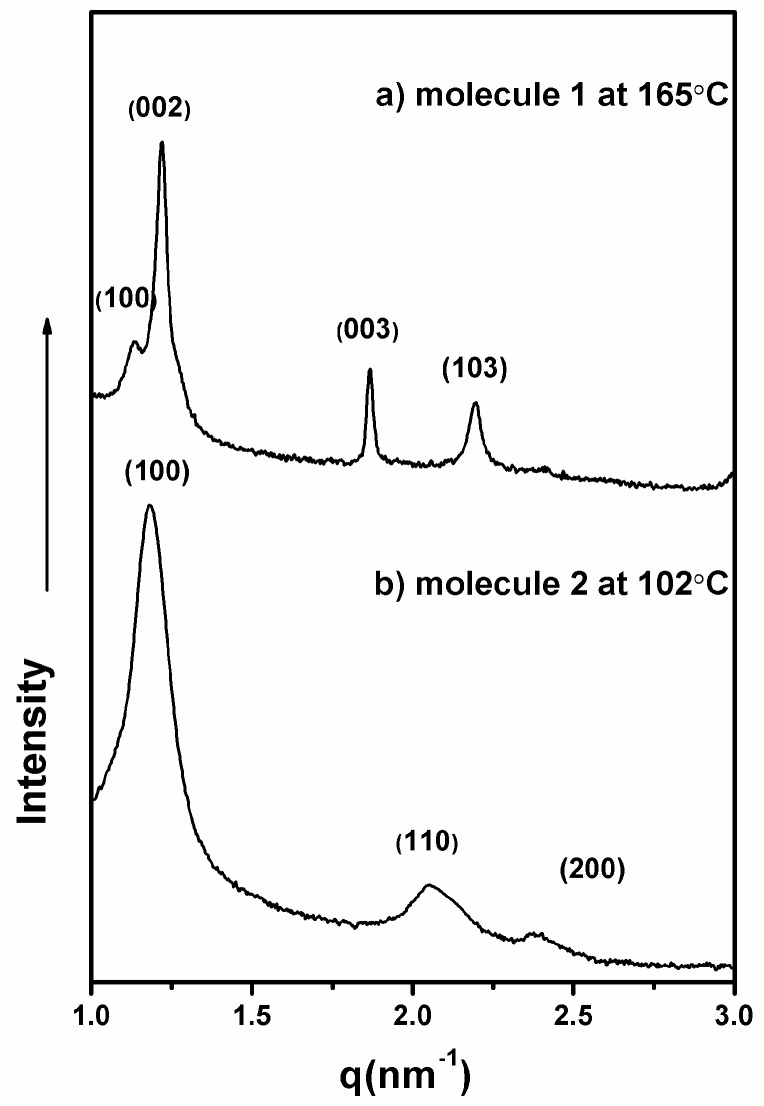
Small-angle X-ray diffraction patterns of **1** and **2** plotted against *q*.

In order to further confirm the ordered nanostructures of **1** and **2**, the SAXS experiment was performed at various temperatures. The SAXS patterns of **1** at 165 °C show four sharp reflections, which can be indexed as the (100), (002), (003), (103) reflections of a 2-D hexagonal perforated lamellar phase with lattice parameters a = 6.39 nm, c = 10.33 nm (Figure 3a). Similarly, in the crystalline phase, the small-angle X-ray diffraction patterns of **2** measured at 102 °C show a number of sharp reflections (Figure 3b), which can be indexed as (100), (110), and (200), which can be described as a hexagonal columnar structure with a lattice constant a = 6.32 nm.

**Figure 4:**
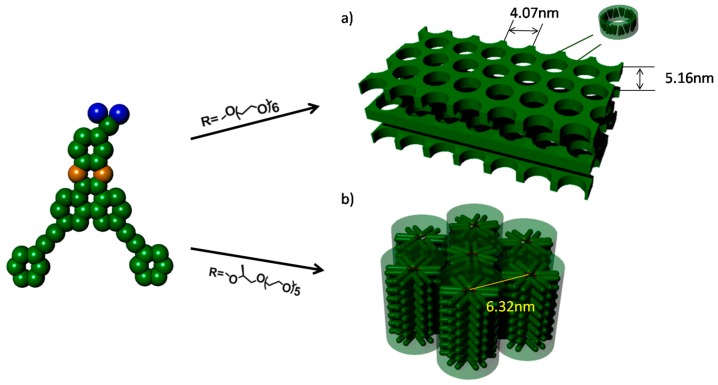
Schematic representation of self-assembly of **1**, **2** in bulk state. (**a**) A hexagonal perforatedlamellar structure of **1**; (**b**) A hexagonal columnar structure of **2**, PEO chains are omitted for clarity.

It can be seen that molecules **1** and **2** have identical lengths of PEO chains and rod segments combined with dibenzo[*a*,*c*]phenazine and phenyl groups, but different lateral groups at the surface of the rod and the coil building block. Although, the structures of molecules **1** and **2** are similar, molecule **1**, with a non-lateral group, self-organizes into honeycomb-like supramolecular nano-structures, while molecule **2**, with a methyl group at the surface of the coil chain, self-assembles into a hexagonal columnar structure in the solid state. This result can be reasonably explained by the strength of the driving force of self-assembly. The methyl group at the coil chains reduces the intermolecular π–π stacking interaction that causes the relatively loose arrangement of the rod building blocks. This, in turn, affects molecular assembly, forming various supramolecular nano-structures when compared to the corresponding molecules with non-methyl groups. In addition, we have reported that molecules with non-carboxyl groups at the 11 position of dibenzo[*a*,*c*]phenazine, compared to molecule **1**, spontaneously self-organize into columnar supramolecular nanostructures. From this result, combined with the above-mentioned data for molecule **1**, we deduce that the synergistic effect of non-covalent hydrogen bonding and π–π stacking interactions of molecule **1** leads to the close-packing of molecule **1**, forming honeycomb-like supramolecular nano-structures (Figure 4). 

**Figure 5:**
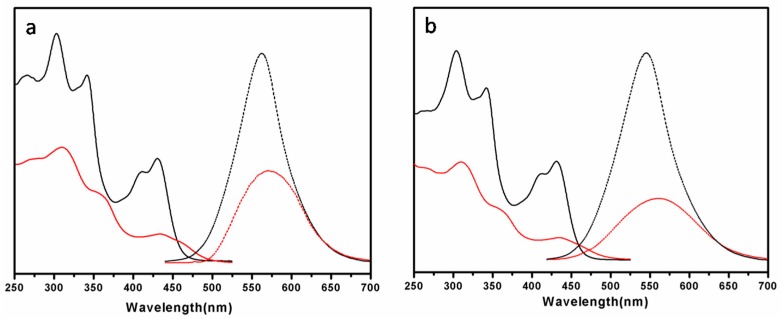
Absorption spectra (dash) and emission spectra (solid) of (**a**) **1**, and (**b**) **2** in CH_2_Cl_2_ (black) and in aqueous solution (red), with 0.01 wt %.

Molecules **1**–**4** are amphipathic; when they are dissolved in water, they can self-assemble into ordered aggregates. Hence, we examined the self-assembling behavior of these molecules in aqueous solution by means of UV-VIS, FL, AFM, and TEM. Figure 5 shows the absorption spectra of **1** and **2** in aqueous solution. We observed a red-shift in the absorption maxima of the aqueous solutions (0.01 wt %) of **1** and **2**, in comparison to those for the dichloromethane solutions. The fluorescence spectrum of **1** in dichloromethane solution (0.01 wt %) exhibits a strong emission maximum at 562 nm (Figure 5a), whereas the emission maximum in aqueous solutions are slightly red-shifted with respect to those observed in the dichloromethane solutions. A significantly quenched fluorescence was then observed, because of the arrangement of conjugated rod building blocks. For self-assembly of **2**–**4**, as shown in Figure 5b and Figure S10 (in Supplementary Materials), similar spectral changes were also exhibited for UV-VIS and FL in diverse solvents, implying that these molecules aggregate into nanoassemblies. To confirm this result, we carried out DLS experiments. The average hydrodynamic diameters of 8.9–288 nm for molecules **1**–**4** were observed, which is indicative of the creation of nanoassembies (see Supplementary Materials, Figure S11).

**Figure 6:**
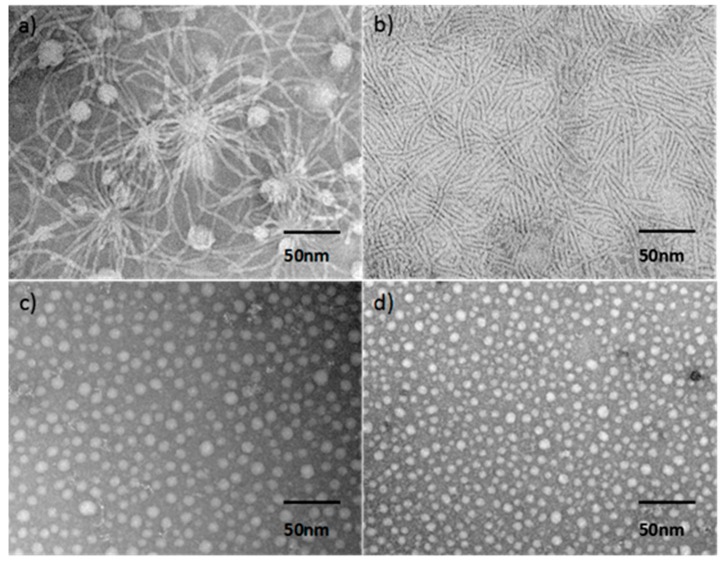
TEM images of (**a**) **1**, (**b**) **2**, (**c**) **3**, and (**d**) **4**, obtained from 0.01 wt % in aqueous solution, samples are negatively stained with uranyl acetate.

To confirm the aggregation structure in aqueous solution, we performed transmission electron microscopy experiments. The samples of molecules **1**–**4** were all cast from an aqueous solution (0.01 wt %), and then negatively stained with uranyl acetate. The micrograph of **1** showed longer nanofibers with a uniform diameter of ~8 nm, and lengths of several micrometers (Figure 6a). Based on the Corey-Pauling-Koltun (CPK) molecular model, the molecular length of the fully extended molecule **1** was estimated to be 4.2 nm. The measured value was more than one molecule but less than twice the molecular length, indicating that the core of the longer nanofibers consisted of aromatic segments in aqueous solution, which are fully interdigitated with each other. Interestingly, molecule **2**, with the same length of the PEO chain, but with methyl groups between the rod and coil segments, showed short nanofibers with lengths of up to ~70 nm and a uniform diameter of ~8 nm (Figure 6b). This can be explained by the presence of a methyl group at the surface of the rod and coil segments, causing the aromatic segments to be packed more loosely; subsequently, this leads to the formation of shorter fibers compared to **1** in aqueous solution. Therefore, we determined that the short fiber forms from the aromatic segment were fully interdigitated with each other. TEM investigation of **3** and **4** with rod and coil segments revealed that molecules spontaneously self-assemble into micellar and relatively big spherical aggregates with a diameter of ranging from ~11 to ~10 nm (Figure 6c,d), which can be observed from the DLS results. Further distinct evidence of self-assembling morphologies was obtained from the AFM images of molecules **1**–**4**, were are in accord with the TEM results of these molecules (see Supplementary Materials, Figure S12).

**Figure 7:**
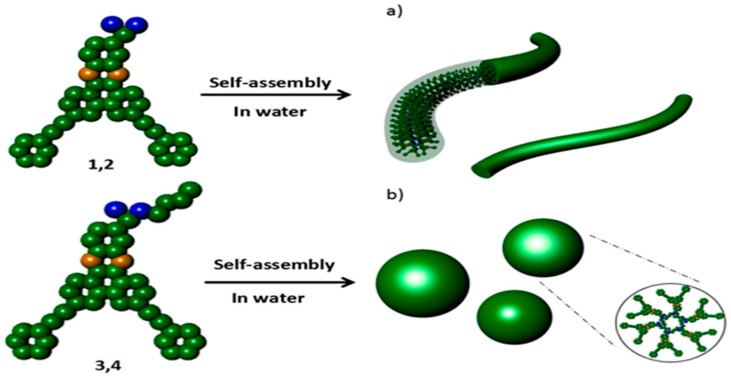
Schematic representation of the proposed self-assembly of molecules in aqueous solution: (**a**) nanofibers for **1** and **2**; (**b**) micelle or nanoparticles for **3** and **4**, PEO chains are omitted for clarity.

Similar to self-assembly in bulk state, molecules **1**–**4**,which have an identical rod and coil building block, apart from the lateral groups at the surface of rod and coil segments or at the 11 position of the dibenzo[*a*,*c*]phenazine unit, exhibit different assembly morphologies and aggregate sizes in their self-assembling process. Molecules **1** and **2** incorporate carboxyl groups and, with a non-lateral group or with a methyl group at the surface of the coil chain, are able to self-assemble into long or short nanofibers, respectively. However, molecules **3** and **4**, which incorporate butoxy carbonyl groups, self-assemble into micelles or nanoparticles in aqueous solution. This can be interpreted visually from the synergistic interaction between the π–π stacking of rod segments and the hydrophilic-hydrophobic effect of molecules **1**–**4**. Molecules **3** and **4**, with a bulky butoxycarbonyl group, have a stronger lateral steric effect than molecules **1** and **2**, which have a small lateral carboxyl group. Subsequently, it is able to cause the loose packing of molecules, forming micelles, and nanoparticles composed with aggregated micelles, through lower intermolecular interaction, when compared to molecules **1** and **2**. In short, by tuning the molecular interaction and hydrophilic and hydrophobic interaction, the curve of the hydrophilic coil segment and hydrophobic rigid segment could be controlled. This led to the production of diverse ordered supramolecular nanoassemblies (Figure 7).

## 4. Conclusions

Amphiphilic rod-coil molecules composed of phenyl, dibenzo[*a*,*c*]phenazine units and PEO coil chains were successfully synthesized. The aggregation behavior of these amphiphiles was investigated in both crystalline phase and aqueous solution. Molecule **1** was found to self-assemble into a hexagonal perforated lamellar structure in the crystalline phase. In contrast, molecule **2**, which incorporated a lateral methyl group at the surface of the rod and coil segments, spontaneously self-organized into hexagonal columnar structures in the bulk state. In aqueous solution, **1** and **2**, with a carboxyl group at the 11 position of dibenzo[*a*,*c*]phenazine, self-assembled into long and short nanofibers, respectively. Whereas, molecules **3** and **4**, with butoxycarbonyl groups at the rod building block, self-organized into spherical aggregates. The experimental results demonstrate that slight changes of molecular structure can lead to the formation of various morphological supramolecular aggregates, by tuning the intermolecular interaction of the amphiphilic rod-coil molecular system. This is of great significance to the study of advanced functional materials for drug-delivery systems and biomolecular sensors.

## Figures and Tables

**Table 1:** Thermal transitions of molecules **1** and **2** (data are from the second heating and the first cooling scans).

Molecule	*f*coil_a_	Phase transition (°C)	
		Heating	Cooling
1	0.53	HPL177i	i159HPL
2	0.54	Col_h_115i	i120Col_h_
3	0.50	——	——
4	0.52	——	——

a = volume fraction of coil to rod-coil molecule [39]. (HPL: hexagonal perforated lamella; i: isotropic phase; Col_h_: hexagonal columnar).

## Supplementary Materials

The following are available online at www.mdpi.com/2073-4360/9/12/685/s1, Figure S1: ^1^H NMR spectrum of molecule **1** in CDCl_3_; Figure S2: ^1^H NMR spectrum of molecule **2** in CDCl_3_; Figure S3: ^1^H NMR spectrum of molecule **3** in CDCl_3_; Figure S4: ^1^H NMR spectrum of molecule **4** in CDCl_3_; Figure S5: MALDI-TOF-MS spectrum of molecule **1** (matrix: CHCA); Figure S6: MALDI-TOF-MS spectrum of molecule **2** (matrix: CHCA); Figure S7: MALDI-TOF-MS spectrum of molecule **3** (matrix: CHCA); Figure S8: MALDI-TOF-MS spectrum of molecule **4** (matrix: CHCA); Figure S9: TGA of molecule **1**; Figure S10: Abosorption spectra (dash) and emission spectra (solid) of (**a**) **3**, (**b**) **4** in CH_2_Cl_2_ (black) and in an aqueous solution (red) with 0.01 wt %; Figure S11: Size distribution graphs of molecule **1**–**4** in aqueous solutions (0.01 wt %); Figure S12: AFM images of molecule (**a**) **1**, (**b**) **2**,(**c**) **3** and (**d**) **4** from 0.01 wt % aqueous solution; Table S1: Small-angle X-ray diffraction data for hexagonal perforated lamella for molecule **1** (measured at 165 °C); Table S2: Small-angle X-ray diffraction data for hexagonal columnar **2** (measured at 102 °C).

## References

[1] Willersinn J., Schmidt B.V.K.J. (2017). Self-Assembly of Double Hydrophilic Poly(2-ethyl-2-oxazoline)-*b*-poly(*N*-vinylpyrrolidone) Block Copolymers in Aqueous Solution. Polymers.

[2] Egli S., Schlaad H., Bruns N., Meier W. (2011). Functionalization of Block Copolymer Vesicle Surfaces. Polymers.

[3] Wei G., Su Z., Reynolds N.P., Arosio P., Hamley I.W., Gazit E., Mezzenga R. (2017). Self-assembling Peptide and Protein Amyloids: From Structure to Tailored Function in Nanotechnology. Chem. Soc. Rev..

[4] Busseron E., Ruff Y., Moulin E., Giuseppone N. (2013). Supramolecular Self-assemblies as Functional Nanomaterials. Nanoscale.

[5] He T., Tang T., Lin C., Shen X., Lu C., Xu L., Gu Z., Xu Z., Qiu H., Zhang Q. (2017). Conjugated Polymers Containing BODIPY and Fluorene Units for Sensitive Detection of CN^−^ Ions: Site-Selective Synthesis, Photo-Physical and Electrochemical Properties. Polymers.

[6] Kim H.J., Liu F., Ryu J.H., Kang S.K., Zeng X.B., Ungar G., Lee J.K., Zin W.C., Lee M. (2012). Self-Organization of Bent Rod Molecules into Hexagonally Ordered Vesicular Columns. J. Am. Chem. Soc..

[7] Sugimoto S., Oda Y., Hirata T., Matsuyama R., Matsunob H., Tanak K. (2017). Surface Segregation of a Branched Polymer with Hydrophilic Poly[2-(2-ethoxy)ethoxyethyl Vinyl Ether] side Chains. Polym. Chem..

[8] Higashihara T., Liu C.L., Chen W.C., Ueda M. (2011). Synthesis of Novel π-Conjugated Rod-Rod-Rod Triblock Copolymers Containing Poly(3-hexylthiophene) and Polyacetylene Segments by Combination of Quasi-Living GRIM and Living Anionic Polymerization. Polymers.

[9] Yang Y., Cui J., Li Z., Zhong K., Jin L.Y., Lee M. (2016). Self-Assembly of n-Shaped Rod-Coil Molecules into Thermoresponsive Nanoassemblies: Construction of Reversible Helical Nanofibers in Aqueous Environment. Macromolecules.

[10] Yu Y.Y., Chien W.C., Tsai C.L. (2017). Preparation and Characterization of Thermo-Responsive Rod-Coil Diblock Copolymers. Polymers.

[11] Yu S., Yang Y., Chen T., Xu J.Z., Jin L.Y. (2017). Donor–Acceptor Interaction-Driven Self-Assembly of Amphiphilic Rod–Coil Molecules into Supramolecular Nanoassemblies. Nanoscale.

[12] Li Z., Yang Y., Chen T., Jin L.Y., Lee M. (2016). Construction of Supramolecular Assemblies from Self-Organization of Amphiphilic Molecular Isomers. Chem. Asian J..

[13] Lim Y., Moon K.S., Lee M. (2008). Rod-coil Block Molecules: Their Aqueous Self-assembly and Biomaterials Applications. J. Mater. Chem..

[14] Poppe S., Poppe M., Ebert H., Prehm M., Chen C., Liu F., Werner S., Bacia K., Tschierske C. (2017). Effects of Lateral and Terminal Chains of X-Shaped Bolapolyphiles with Oligo(phenyleneethynylene) Cores on Self-Assembly Behaviour. Part 1: Transition between Amphiphilic and Polyphilic Self-Assembly in the Bulk. Polymers.

[15] Guan Z., Wang L., Zhua X., Lin J. (2017). Striped Patterns Self-assembled from Rod–Coil Diblock Copolymers on Spherical Substrates. Mater. Chem. Front..

[16] Takenami K., Uemura S., Funahashi M. (2016). In situ Polymerization of Liquid-crystalline thin Films of electron-transporting Perylene Tetracarboxylic Bisimide bearing Cyclotetrasiloxane Rings. RSC Adv..

[17] Oyafuso M.H., Carvalho F.C., Takeshita T.M., Souza A.L.R., Araújo D.R., Merino V., Gremião M.P.D., Chorilli M. (2017). Development and In Vitro Evaluation of Lyotropic Liquid Crystals for the Controlled Release of Dexamethasone. Polymers.

[18] Lee M., Lee S., Jiang L.H. (2004). Stimuli-Responsive Supramolecular Nanocapsules from Amphiphilic Calixarene Assembly. J. Am. Chem. Soc..

[19] Lee E., Kim J.K., Lee M. (2009). Tubular Stacking of Water-Soluble Toroids Triggered by Guest Encapsulation. J. Am. Chem. Soc..

[20] Kim H.J., Kim T., Kim T., Lee M. (2011). Responsive Nanostructures from Aqueous Assembly of Rigid−Flexible Block Molecules. Acc. Chem. Res..

[21] Huang Z., Ryu J.H., Lee E., Lee M. (2007). Tunable Columnar Organization by Twisted Stacking of End-Capped Aromatic Rods. Chem. Mater..

[22] Chen S., Ma C., Huang Z., Lee M. (2014). Controlled Helicity of the Rigid-Flexible Molecular Assembly Triggered by Water Addition: From Nanocrystal to Liquid Crystal Gel and Aqueous Nanofibers. J. Phys. Chem. C.

[23] Chen X., He Y., Kim Y., Lee M. (2016). Reversible, Short α-Peptide Assembly for Controlled Capture and Selective Release of Enantiomers. J. Am. Chem. Soc..

[24] Zhuang Z., Cai C., Jiang T., Lin J., Yang C. (2014). Self-assembly Behavior of rod–coil–rod polypeptide block copolymers. Polymer.

[25] Xia Y., Chen J.Z., Sun Z.Y., Shi T.F., An L.J., Jia Y.X. (2010). Self-assembly of linear ABC coil-coil-rod triblock copolymers. Polymer.

[26] Gorecka E., Pociecha D., Mieczkowski J., Matraszek J., Guillon D., Donnio B. (2004). Supramolecular Self-Assembly into Biofunctional Soft Nanotubes: From Bilayers to Monolayers. J. Am. Chem. Soc..

[27] Gimeno N., Ros M.B., Serrano J.L., Fuente M.R.D. (2008). Axially Polar Columnar Phase Made of Polycatenar Bent-Shaped Molecules. Chem. Mater..

[28] Dantlgraber G., Shen D., Diele S., Tschierske C. (2002). Antiferroelectric Switchable Mesophases of Nonchiral Bent-Core Liquid Crystals Containing Fluorinated Central Cores. Chem. Mater..

[29] Keith C., Reddy R.A., Baumeister U., Tschierske C. (2004). Banana-Shaped Liquid Crystals with Two Oligosiloxane End-Groups:  Field-Induced Switching of Supramolecular Chirality. J. Am. Chem. Soc..

[30] Shen D., Pegenau A., Diele S., Wirth I., Tschierske C. (2000). Molecular Design of Nonchiral Bent-Core Liquid Crystals with Antiferroelectric Properties. J. Am. Chem. Soc..

[31] Keith C., Reddy R.A., Hauser A., Baumeister U., Tschierske C. (2006). Silicon-Containing Polyphilic Bent-Core Molecules: The Importance of Nanosegregation for the Development of Chirality and Polar Order in Liquid Crystalline Phases Formed by Achiral Molecules. J. Am. Chem. Soc..

[32] Keith C., Dantlgraber G., Reddy R.A., Baumeister U., Tschierske C. (2007). Ferroelectric and Antiferroelectric Smectic and Columnar Liquid Crystalline Phases Formed by Silylated and Non-Silylated Molecules with Fluorinated Bent Cores. Chem. Mater..

[33] Zhang Y., Baumeister U., Tschierske C., O’Callaghan M.J., Walker C. (2010). Achiral Bent-Core Molecules with a Series of Linear or Branched Carbosilane Termini: Dark Conglomerate Phases, Supramolecular Chirality and Macroscopic Polar Order. Chem. Mater..

[34] Wang Z., Cui J., Liang Y., Chen T., Lee M., Yin B., Jin L.Y. (2013). Supramolecular Nanostructures from Self-Assembly of T-Shaped Rod Building Block Oligomers. Polym. Chem..

[35] You S., Zhong K., Jin L.Y. (2017). Control of supramolecular nanoassemblies by tuning the interactions of bent-shaped rod–coil molecules. Soft Matter.

[36] Lu Z., Zhong K., Liu Y., Li Z., Chen T., Jin L.Y. (2016). Self-organizing *p*-quinquephenyl Building Blocks Incorporating Lateral Hydroxyl and Methoxyl Groups into Supramolecular Nano-assemblies. Soft Matter.

[37] Liu Y., Zhong K.L., Li Z.H., Wang Y.Q., Chen T., Lee M., Jin L.Y. (2015). Synthesis and Self-assembly of Amphiphilic Bent-shaped Molecules Based on Dibenzo[*a*,*c*]phenazine and Poly(ethylene oxide) Units. Polym. Chem..

[38] Li Z., Wu Z., Mo G., Xing X., Liu P. (2014). A Small-angle X-ray Scattering Station at Beijing Synchrotron Radiation Facility. Instrum. Sci. Technol..

[39] Lee M., Cho B.K., Jang Y.G., Zin W.C. (2000). Spontaneous Organization of Supramolecular Rod-Bundles into a Body-Centered Tetragonal Assembly in Coil–Rod–Coil Molecules. J. Am. Chem. Soc..

[40] Cai Y., Zheng M., Zhu Y., Chen X.F., Li C.Y. (2017). Tunable Supramolecular Hexagonal Columnar Structures of Hydrogen-Bonded Copolymers Containing Two Different Sized Dendritic Side Chains. ACS Macro Lett..

[41] Cristiano R., Eccher J., Bechtold I.H., Tironi C.N., Vieira A.A., Molin F., Gallardo H. (2012). Luminescent Columnar Liquid Crystals Based on Tristriazolotriazine. Langmuir.

[42] Liu C.Y., Chen H.L. (2017). Undulating the Lamellar Interface of Polymer–Surfactant Complex by Dendrimer. Macromolecules.

[43] Li Z., Fan S.-F., Chen T., Jin L.Y. (2016). Organization of Coil–Rod–Coil Triblock Copolymers into Columnar Aggregates. Acta Polym. Sin..

