# Supplementary material for: Construction of Supramolecular Nanostructures from V-Shaped Amphiphilic Rod-Coil Molecules Incorporating Phenazine Units
Source: Polymers (Basel). 2017 Dec 7;9(12):685. doi: 10.3390/polym9120685 (PMC6419232; doi:10.3390/polym9120685)
Supplement: Supplementary file 1 [file polymers-09-00685-s001.pdf]

Supplementary materials

Construction of Supramolecular Nanostructures from V-shaped Amphiphilic Rod-coil Molecules Incorporating Phenazine Units

Junying Xu [1], Shengsheng Yu [1], Long Yi Jin [1,*] andKeli Zhong [2,*]

1 Key Laboratory for Organism Resources of the Changbai Mountain and Functional Molecules, and Department of Chemistry, College of Science, Yanbian University, Yanji, Jilin, 133002, China

2 College of Chemistry, Chemical Engineering and Food Safety, Bohai University, Jinzhou 121013, China

*Correspondence: lyjin@ybu.edu.cn (L. Y. Jin); zhongkeli2000@126.com (K. Zhong)

1. Experimental Section

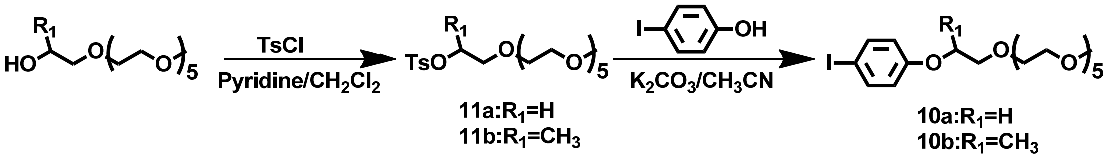

Scheme S1. Synthetic route of molecules **10** and **11**

*1.1 Synthesis of molecules **11a** and **11b***

Molecules **11a** and **11b** were the same as the experimental procedure. **11a** is exemplified as a representative example.The compound poly(ethylene glycol) methyl ether (M_w=296) (12.00 g, 40.50 mmol), TsCl (15.00 g, 78.90 mmol) and pyridine(25mL) were put into 250ml one-neck flask dissolved in dry dichloromethane (60 mL). The mixture was stirred at room temperature for 12h, add distilled water to the reaction solution (60mL), stirring 2h. Then add concentrated hydrochloric acid, significant acidity stirring 2h. The resulting solution was dried ($MgSO_4$) and filtered. The solvent was removed in a rotary evaporator and the crude product was purified by column chromatography on silica gel using CH_2Cl_2:MeOH (20:1 v/v) as eluent to yellow yield (13.50g,74.0%). ^{1}H NMR (300 MHz, $CDCl_3$, δ, ppm): 7.79 (d, J = 3 Hz, 2H), 7.46 (d, J = 6 Hz, 2H), 4.14 (t, J = 5 Hz, 2H), 3.52-3.65 (m, 22H), 3.27 (s, 3H), 2.39 (s, 3H).

*1.2 Synthesis of molecules **10a** and **10b***

Molecules **10a** and **10b** were synthesized using the same procedure. A representative example is described for **10a**. Compound **11a** (0.60 g, 1.30 mmol) and 4-iodophenol (0.38 g, 1.70 mmol) were dissolved in acetonitrile (40 mL) and then excess potassium carbonate (0.92 g, 6.67 mmol) was added to the solution. The mixture was heated at reflux overnight with

vigorous stirring. The solvent was removed in a rotary evaporator and the resulting mixture was poured into water and extracted with ethyl acetate, dried over anhydrous magnesium sulfate and filtered. The solvent was evaporated to dryness. The crude product was purified by column chromatography (silica gel) using ethyl acetate as eluent to yield a slightly yellow liquid (0.49 g, 75.3%). ^{1}H NMR (300 MHz, $CDCl_3$, δ, ppm): 7.69 (d, *J* = 6 Hz, 2H),6.70 (d, *J* = 6 Hz, 2H), 4.31(t, *J* = 6 Hz, 2H), 3.79 (t, *J* = 6 Hz,2H) 3.70-3.51 (m, 20H), 3.30 (s, 3H).

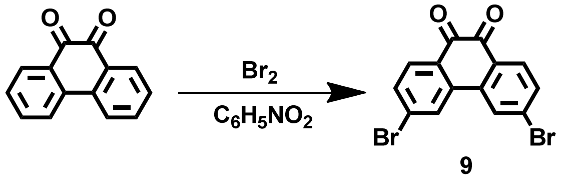

Scheme S2. Synthetic route of molecules **9**

*1.3 Synthesis of molecule **9***

9, 10-Phenanthrenequinone (10.40 g, 50.00 mmol) and BPO (0.80 g, 3.30 mmol) were added to nitrobenzol (90 mL). Liquid bromine (5.80 mL) was introduced with a syringe. The reaction mixture was stirred at 80 ℃ for 36 hours with an environment of high pressure mercury lamp. The resulting mixture was cooled to room temperature until solid separated out and then filtered the mixture. The solid was washed with absoluted ethanol. Absoluted ethanol (20 mL) was added into filtrate and then placed in 0 ℃ until solid separated out. Filtered the mixture and combined the solid. An earthy yellow powder was obtained after dried overnight under vacuum (12.52 g, 68.8%). ^{1}H NMR (300 MHz, $CDCl_3$, δ, ppm): 8.12 (d, *J* = 2 Hz, 2H), 8.07 (d, *J* = 6 Hz, 2H), 7.67 (m, 2H).

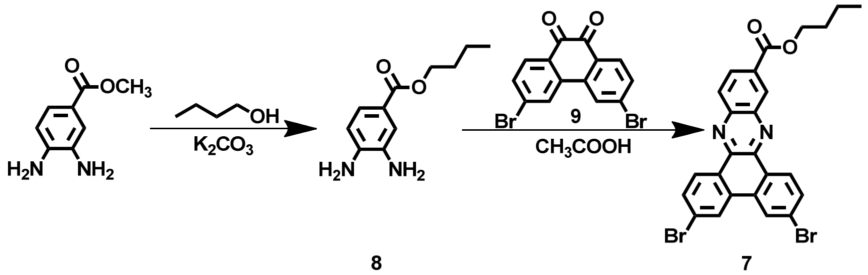

Scheme S3. Synthetic route of molecules **7** and **8**

1.4 Synthesis of molecule 8

3, 4-Diaminobenzoic acid methyl ester (1.00 g, 6.00 mmol) and Potassium carbonate (1.67 g, 12.00mmol) were dissolved in freshly distilled butanol (60ml). The mixture was degassed and then heated at reflux for 24h with vigorous stirring under nitrogen. The solvent was removed in a rotary evaporator and the resulting mixture was poured into water and extracted with dichloromethane, dried over anhydrous magnesium sulfate. The crude product was purified

by column chromatography on silica gel using methylene dichloride as eluent to brown viscous liquid(0.87 g,70%). ^{1}H NMR (300 MHz, $CDCl_3$, δ, ppm): 7.48 (d, J = 6 Hz, 1H), 7.42 (s, 1H), 6.67(d, J = 6 Hz, 1H), 4.25 (t, J = 6 Hz, 2H), 1.72 (t, J = 6 Hz, 3H), 1.47(t, J = 4 Hz, 3H), 0.96(t, J = 6 Hz, 3H).

1.5 Synthesis of molecule **7**

Compound **8** (4.00 g, 24.07 mmol) and compound **9** (3.35 g, 48.14mmol) were dissolved in glacial acetic acid. The reaction mixture was heated to reflux for 7 hours. The resulting mixture was cooled to room temperature and then filtered. Filter cake was washed by glacial acetic acid , then a yellow solid was obtained (5.01 g, 91.4%). ^{1}H NMR (300 MHz, $CDCl_3$, δ, ppm): 9.17 (d, J = 5 Hz, 2H), 8.96 (s, 1H), 8.52(s, 2H), 8.45 (d, J = 6 Hz, 1H), 8.29 (d, J = 6 Hz, 1H), 7.83 (d, J = 6 Hz, 2H), 4.48 (t, J = 5 Hz, 2H), 1.89 (m, 2H), 1.59 (m, 5H), 1.06 (t, J = 4 Hz, 3H).

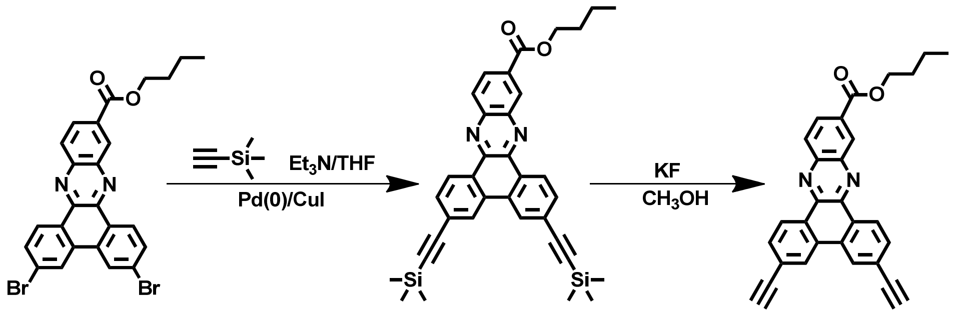

Scheme S4. Synthetic route of molecules **5** and **6**

1.6 Synthesis of molecule **6**

Compound **7** (2.10 g, 3.90 mmol), TMSA (2.30 g, 23.4mmol), ETA (35 ml) were dissolved in dry THF (100 ml). Then Bis(triphenylphosphine)palladium (Ⅱ) chloride (224.90 mg, 1.20 mmol) and CuI (682.30 mg, 0.59 mmol) were added in the dark. The mixture was degassed and then heated at reflux for 24 h with vigorous stirring under nitrogen. The solvent was removed in a rotary evaporator and the resulting mixture was poured into water and extracted with dichloromethane, dried over anhydrous magnesium sulfate. The solid was collected by filtration, and further purified by flash chromatography (5:1 and then 3:1 petroleum ether/dichloromethane) to give 6 as a yellow solid (1.65 g, 73.2%). ^{1}H NMR (300 MHz, $CDCl_3$, δ, ppm): 9.31 (d, J = 5 Hz, 2H), 9.00 (s, 1H), 8.65 (s, 1H), 8.44 (d, J = 6 Hz, 1H), 8.31 (d, J = 5 Hz, 1H), 7.81 (d, J = 4 Hz, 2H), 4.47 (t, J = 5 Hz, 2H), 1.89 (m, 2H), 1.59 (m, 5H), 1.59 (t, J = 5 Hz, 2H), 1.05 (t, J = 6 Hz, 3H), 0.37 (m, 18H).

1.7 Synthesis of molecule **5**

Compound **6** (0.65 g, 1.13mmol) and potassium fluoride (1.32 g, 22.72 mmol) were dissolved in alcohol (100 ml). The reaction mixture was heated to reflux for 20 h. The solvent was removed in a rotary evaporator . Hydrochloric acid (10%) and water were added into the

resulting mixture and filtered. Filter cake was washed by water and obtained a yellow solid. Then the filtrate was extracted with ethyl acetate and dichlormethane, the combined organic layer was dried over anhydrous magnesium sulfate and filtered. The solvent was evaporated to dryness. The crude product and the yellow solid were combined and then purified by column chromatography (silica gel) using petroleum ether/dichloromethane (5:1 and then 3:1 v/v) as eluent to yield a light yellow solid (0.27 g, 56.6%). ^{1}H NMR (300 MHz, $CDCl_3$, δ, ppm): 9.26 (q, *J* = 6.0 Hz, 2H), 8.98 (t, *J* = 3.0 Hz, 1H), 8.6 (s, 2H), 8.44 (q, *J* = 3.0 Hz, 2H), 8.3 (d, *J* = 6 Hz, 2H), 7.82 (q, *J* = 3.0 Hz, 2H), 4.6-4.44 (m, 2H), 3.35 (d, *J* = 3.0 Hz, 2H), 1.86 (t, *J* = 6.0 Hz, 2H), 1.50 (t, *J* = 6.0 Hz, 2H), 1.06(t, *J* = 6.0 Hz, 3H).

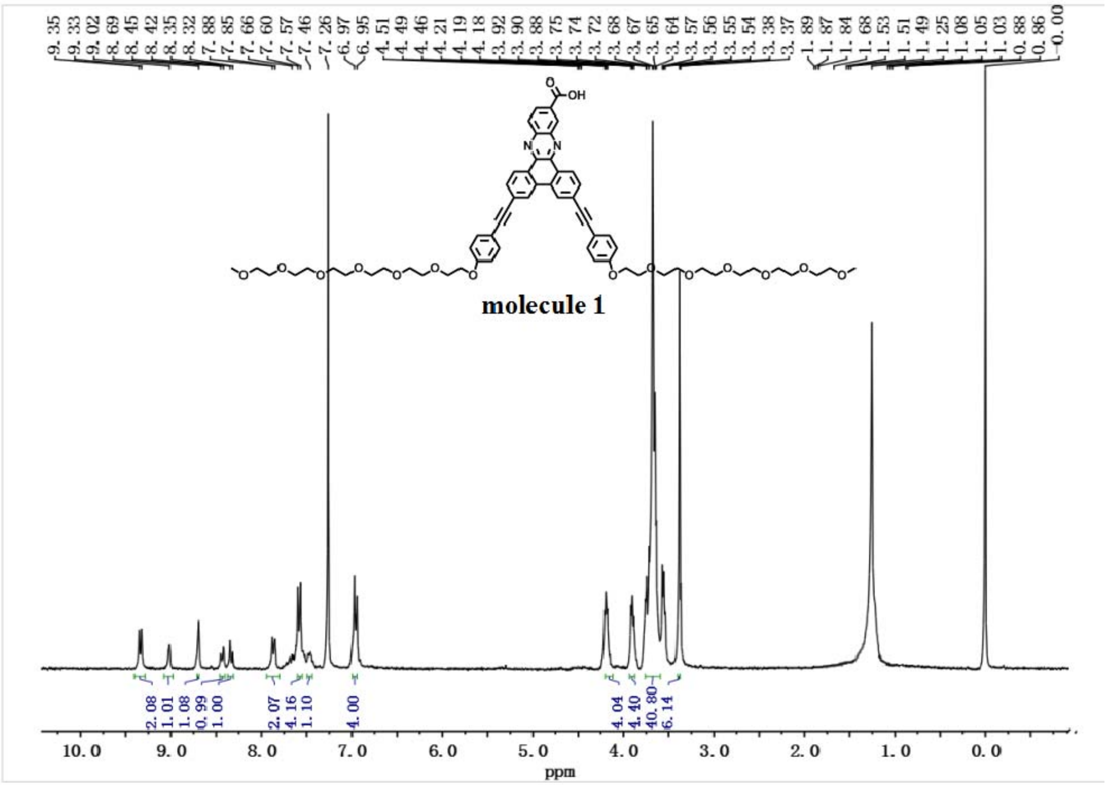

Figure S1. ^{1}H NMR spectrum of molecule **1** in $CDCl_3$.

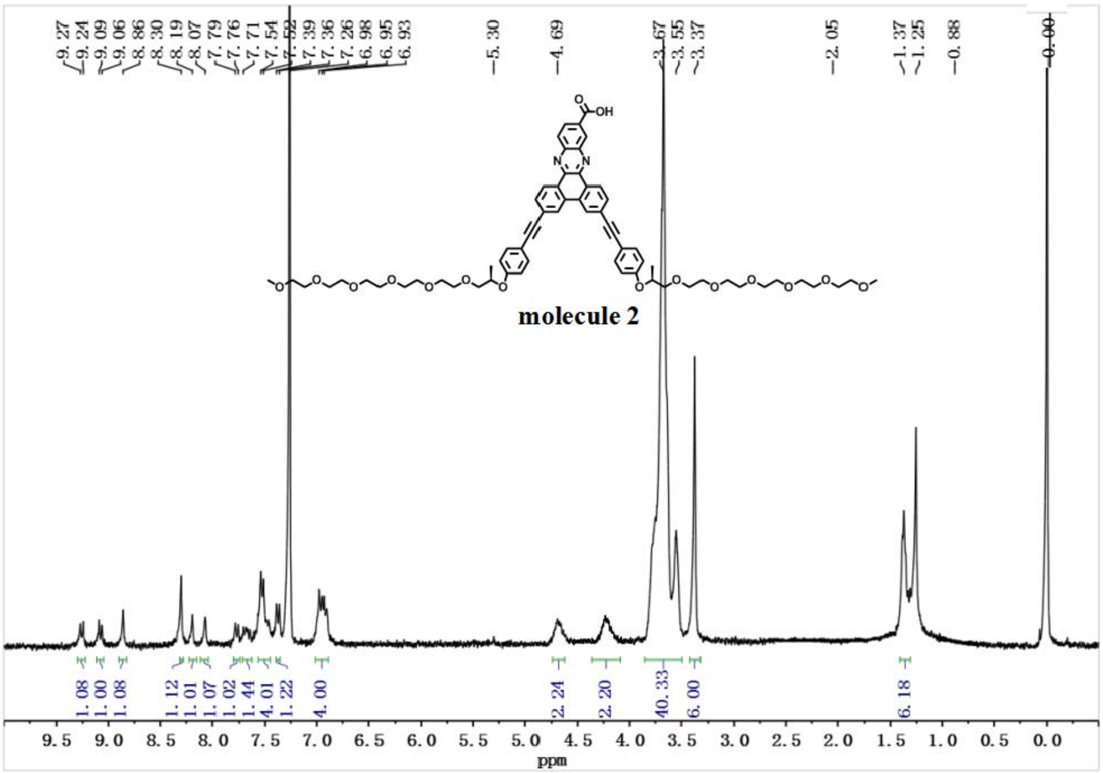

Figure S2. ^{1}H NMR spectrum of molecule **2** in $CDCl_3$.

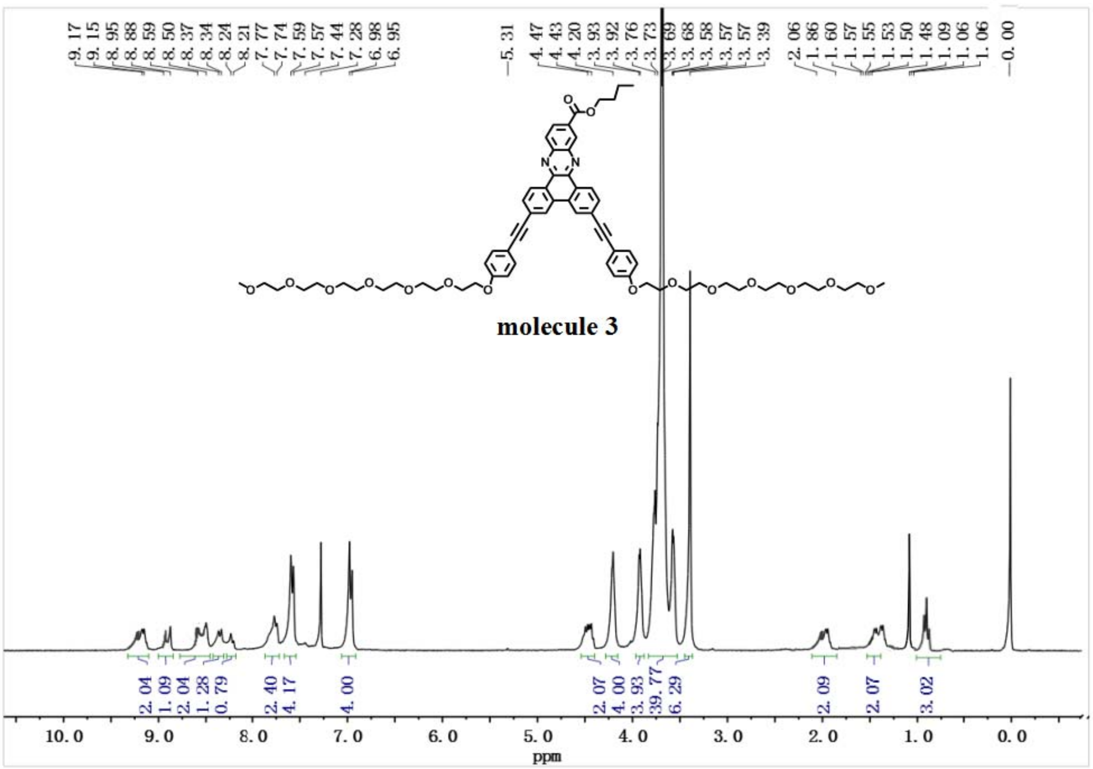

Figure S3. ^{1}H NMR spectrum of molecule 3 in $CDCl_3$.

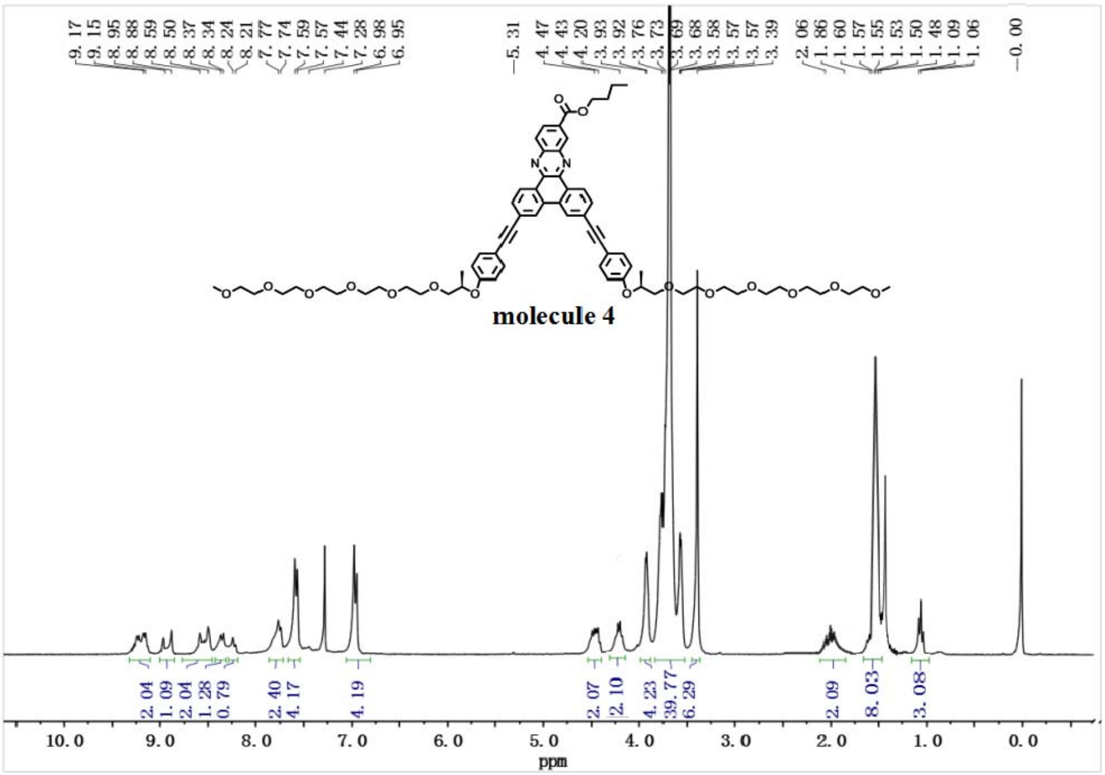

Figure S4. ^{1}H NMR spectrum of molecule 4 in $CDCl_3$.

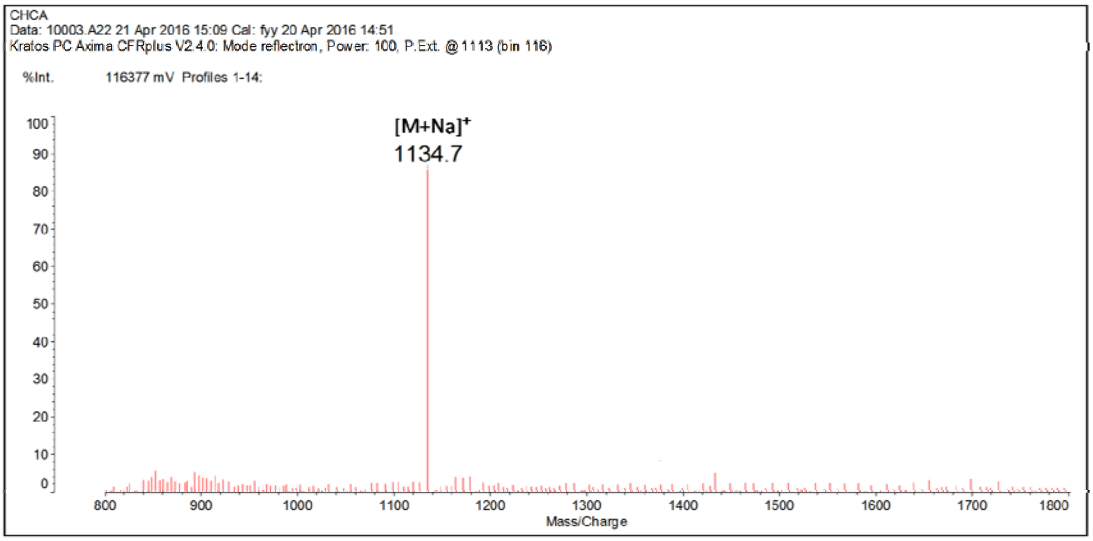

Figure S5. MALDI-TOF-MS spectrum of molecules **1** (matrix: CHCA).

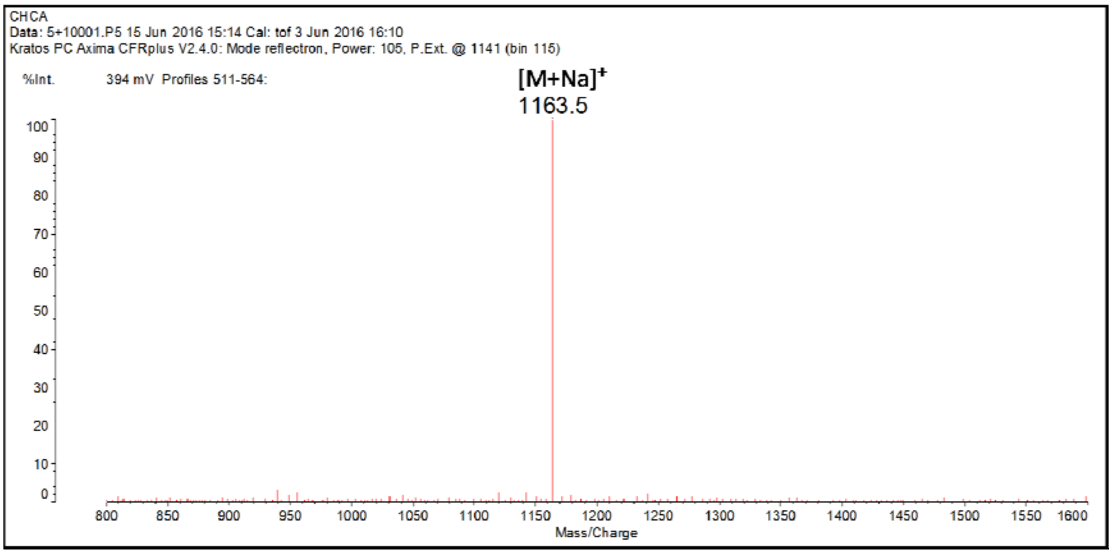

Figure S6. MALDI-TOF-MS spectrum of molecules **2** (matrix: CHCA).

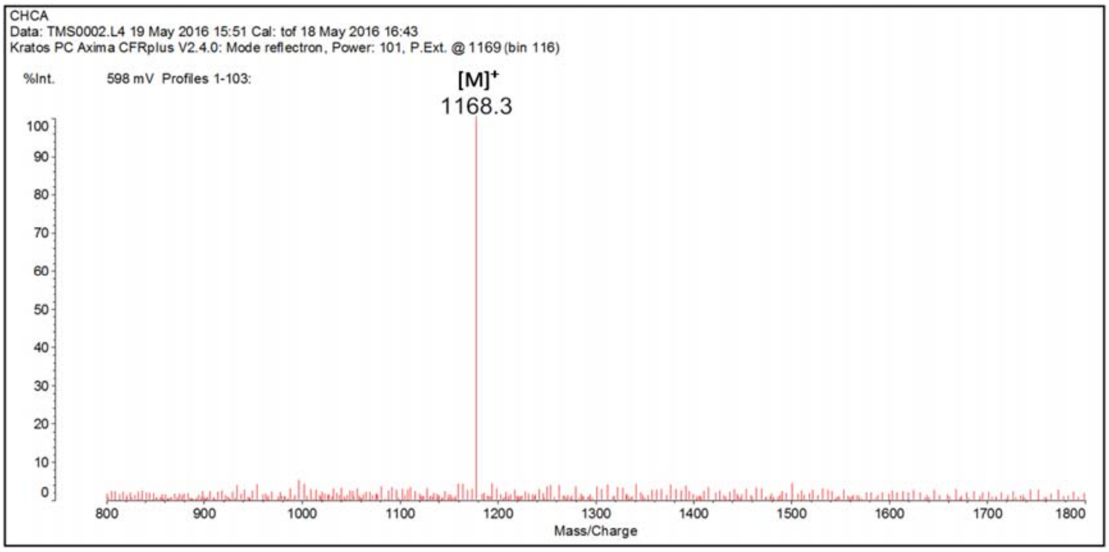

Figure S7. MALDI-TOF-MS spectrum of molecules **3** (matrix: CHCA).

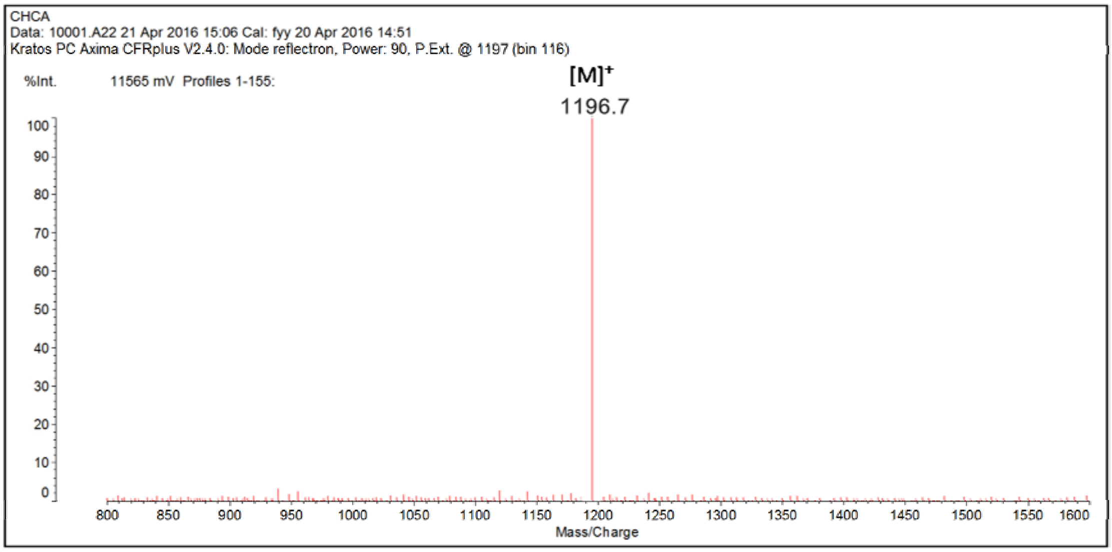

Figure S8. MALDI-TOF-MS spectrum of molecules **4** (matrix: CHCA).

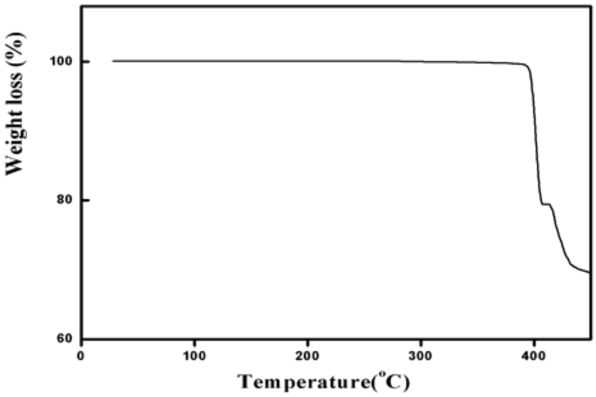

Figure S9. TGA of molecule **1**.

Table S1. Small-angle X-ray diffraction data for hexagonal perforated lamella for molecule **1** (measured at 165°C)

q_{obsd}	q_{calcd}	*h*	*k*	*l*
1.135	1.139	1	0	0
1.216	1.211	0	0	2
1.830	1.823	0	0	3
2.157	2.150	1	0	3

q_{obsd} and q_{calcd} are the scattering vectors of the observed and calculated reflections (λ = 0.154 nm).

Table S2. Small-angle X-ray diffraction data for hexagonal columnar for molecule **2** (measured at 102°C)

q_{obsd}	q_{calcd}	*h*	*k*	*l*
1.181	1.182	1	0	0
2.051	2.051	1	1	0
2.361	2.363	2	0	0

q_{obsd} and q_{calcd} are the scattering vectors of the observed and calculated reflections (λ = 0.154 nm).

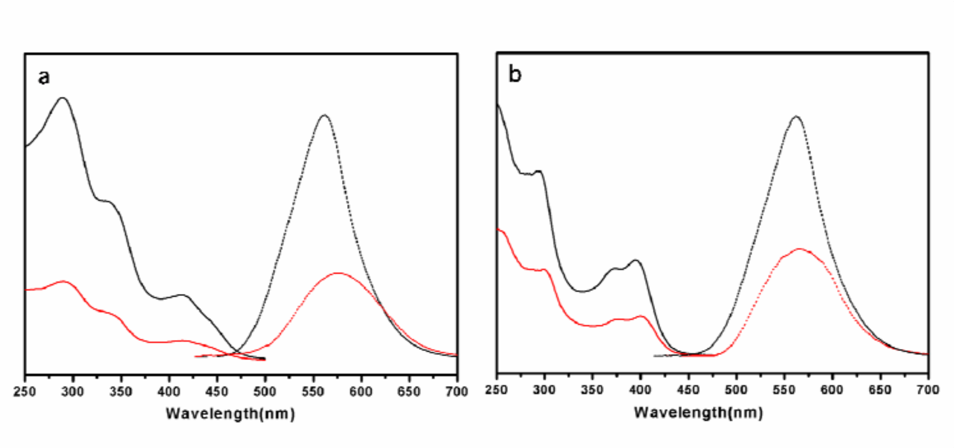

Figure S10. Absorption spectra (dash) and emission spectra (solid) of (**a**)**3**, (**b**)**4** in CH_2Cl_2 (black) and in an aqueous solution (red) with 0.01 wt %.

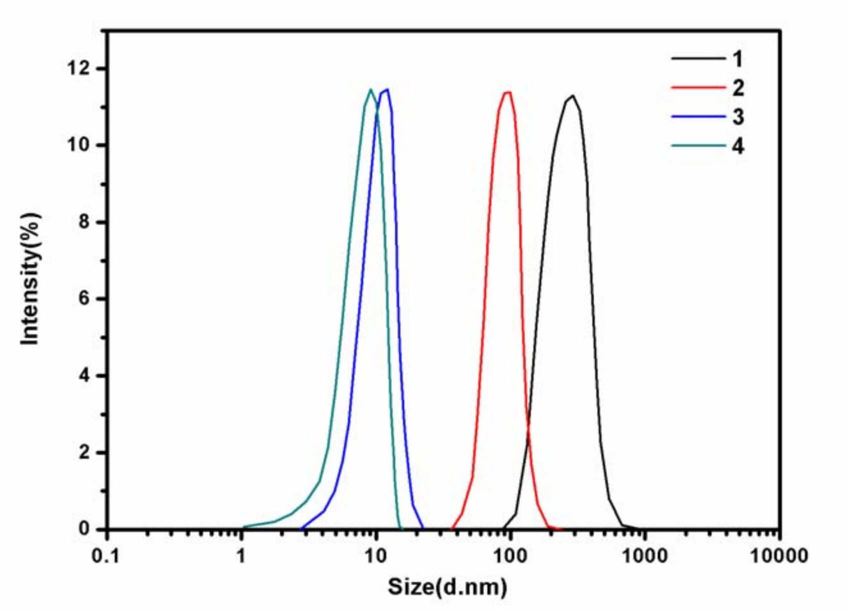

Figure S11. Size distribution graphs of molecules **1-4** in aqueous solutions (0.01 wt %).

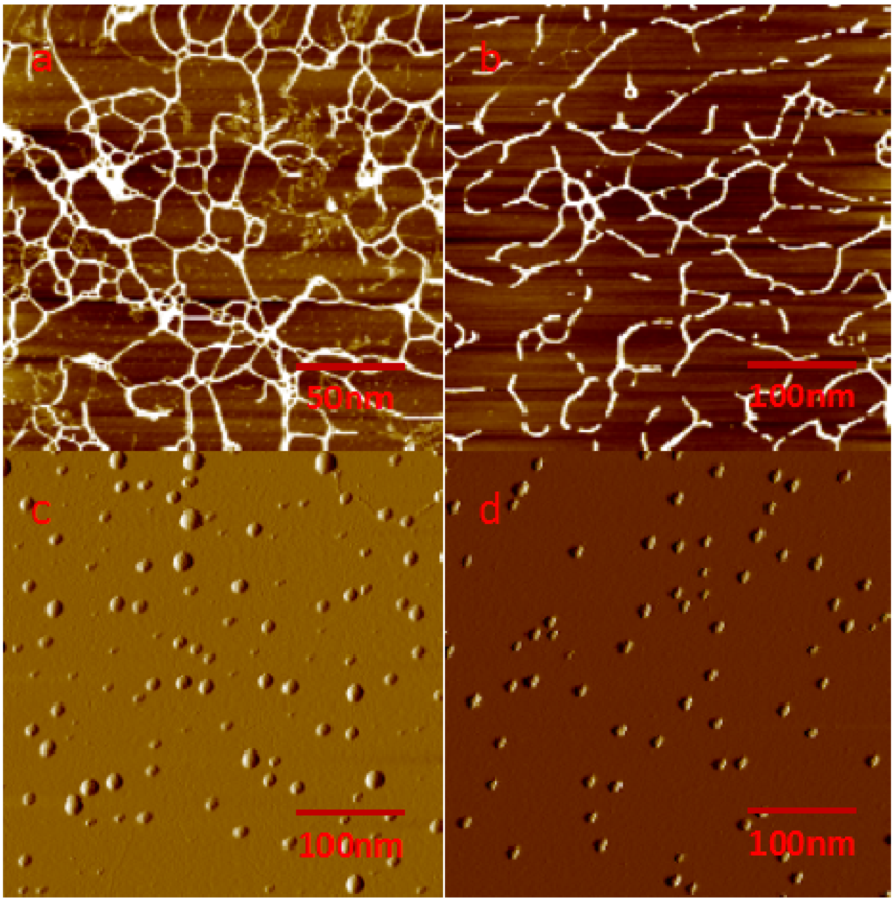

Figure S12. AFM images of molecule (**a**) **1**, (**b**) **2**, (**c**) **3** and (**d**) **4** from 0.01 wt % aqueous solution.
